# Essential Oil Nanoemulsion as Eco-Friendly and Safe Preservative: Bioefficacy Against Microbial Food Deterioration and Toxin Secretion, Mode of Action, and Future Opportunities

**DOI:** 10.3389/fmicb.2021.751062

**Published:** 2021-11-29

**Authors:** Akash Maurya, Vipin Kumar Singh, Somenath Das, Jitendra Prasad, Akash Kedia, Neha Upadhyay, Nawal Kishore Dubey, Abhishek Kumar Dwivedy

**Affiliations:** ^1^Laboratory of Herbal Pesticides, Department of Botany, Institute of Science, Banaras Hindu University, Varanasi, India; ^2^Government General Degree College, Mangalkote, Burdwan, India

**Keywords:** essential oil, biodeterioration, toxins, nanoemulsion, food preservative, eco-friendly

## Abstract

Microbes are the biggest shareholder for the quantitative and qualitative deterioration of food commodities at different stages of production, transportation, and storage, along with the secretion of toxic secondary metabolites. Indiscriminate application of synthetic preservatives may develop resistance in microbial strains and associated complications in human health with broad-spectrum environmental non-sustainability. The application of essential oils (EOs) as a natural antimicrobial and their efficacy for the preservation of foods has been of present interest and growing consumer demand in the current generation. However, the loss in bioactivity of EOs from fluctuating environmental conditions is a major limitation during their practical application, which could be overcome by encapsulating them in a suitable biodegradable and biocompatible polymer matrix with enhancement to their efficacy and stability. Among different nanoencapsulated systems, nanoemulsions effectively contribute to the practical applications of EOs by expanding their dispersibility and foster their controlled delivery in food systems. In line with the above background, this review aims to present the practical application of nanoemulsions (a) by addressing their direct and indirect (EO nanoemulsion coating leading to active packaging) consistent support in a real food system, (b) biochemical actions related to antimicrobial mechanisms, (c) effectiveness of nanoemulsion as bio-nanosensor with large scale practical applicability, (d) critical evaluation of toxicity, safety, and regulatory issues, and (e) market demand of nanoemulsion in pharmaceuticals and nutraceuticals along with the current challenges and future opportunities.

## Introduction

Nowadays, global food safety is one of the major public health issues. Presently, most of the food products *viz*. bakery, dairy, meat, fruits, and vegetables are heavily contaminated due to the presence of various toxigenic species of bacteria and fungi and their associated toxins leading to severe human illness and deaths. Therefore, consumers are demanding healthy, high-quality, and safer food products. Moreover, in tropical and subtropical regions, contamination of food items by various microbes and their associated toxic metabolites not only deteriorates the food but can also alter the nutritional qualities (Barba et al., 2017; Chaudhari et al., 2021a). Consequently, consumption patterns are changing toward the intake of healthy food products with preserved nutritional values (Güneş and Turan, 2017). Therefore, special attention has been given to improve the quality, safety, and security of food systems against microbial spoilage and their associated toxins.

In order to control bacterial and fungal growth, various synthetic preservatives have been commonly applied. However, indiscriminate utilization of these preservatives may cause several negative perceptions in terms of their toxicity to non-target organisms, development of microbial resistance, and degradation of environmental sustainability (Calvo et al., 2017). Therefore, to ensure microbial food safety, contemporary consumers are demanding “safer alternatives” with a green image and possible non-toxic effects on humans and animals (Castro-Rosas et al., 2017; Das et al., 2021c). Currently, plant essential oils (EOs) and components are garnering more attention in the commercial food sector due to their unique aroma, flavors, and antimicrobial properties without affecting organoleptic and nutritional attributes of food items (Burt, 2004). EOs are secondary metabolites of aromatic plants and are considered under the ‘generally recognized as safe’ (GRAS) label by the US- FDA (Ju et al., 2018). The major implication of EOs for the preservation of food commodities along with other chemical components lies in the possible synergistic effect during long term preservation (Jiang et al., 2009). However, applications of EOs are still restricted due to intense aroma, low water solubility, and less stability in fluctuating environmental conditions such as temperature, light, and oxygen (Maurya et al., 2021; Singh et al., 2021). Moreover, some of the EOs only perform their antimicrobial efficacy at higher concentrations leading to negative impact on the organoleptic properties of food products (Olatunde and Benjakul, 2018). Therefore, nanoencapsulation of EOs into different carrier matrices has been regarded as a novel green strategy to overcome the drawbacks with improvement in EOs functionalities and their practical application in food industries (Salvia-Trujillo et al., 2015a; Chaudhari et al., 2021b).

Hydrophobic EOs and their components may be encapsulated into suitable biopolymers to make them more available in watery environments as well as to enhance the antimicrobial efficacy of EOs by increasing uniform distribution on food surfaces (Salvia-Trujillo et al., 2015b; Das A. K. et al., 2020; Lugani et al., 2021). Different nanoencapsulation systems like nanoemulsion, solid lipid nanoparticles, nanofibers, liposomes, and edible films are available with practical utility in food preservation (Aswathanarayan and Vittal, 2019). Among them, nanoemulsion has been considered as more effective (Anwer et al., 2014) and widely used nanometric systems due to at least one dimension less than 100 nm (Hasan et al., 2020; Amiri et al., 2021). Consequently, nanoencapsulation has some remarkable advantages over other encapsulation systems for certain applications such as improved stability of encapsulated active compounds, large surface area to volume ratio, higher bioavailability, mass transfer behavior (Zhang et al., 2021), enhanced bioactivity (Donsì and Ferrari, 2016), and better diffusion to target food systems (McClements et al., 2021). Recently, extensive investigations have been performed on the EOs encapsulation and their potential application in the food industry (Dwivedy et al., 2018; Pabast et al., 2018; Aswathanarayan and Vittal, 2019; Zhu et al., 2020; Chaudhari et al., 2021a). However, to date, there is a lack of knowledge about the utilization of EOs nanoemulsion against microbial contamination, deterioration, and toxin secretion in food for long-term sustainable preservation. Therefore, the present review has briefly discussed (a) processes for the fabrication of nanoemulsion with critical analyses for antibacterial and antifungal activity, (b) diverse mechanisms associated with antimicrobial activity, (c) potential practical applications, (d) the toxicity and safety of nanoemulsions, and (e) future perspectives addressing the research gaps and current challenges.

## Microbial Contamination of Foods

A number of investigations have explained that food commodities, especially vegetables, fruits, meat, and other high fat-containing products are maximally spoiled or wasted due to infection of bacterial pathogens such as *Listeria* spp., *Escherichia* spp., *Campylobacter* spp., *Bacillus* spp., *Salmonella* spp., and *Klebsiella* spp. (Hadian et al., 2017; Kawacka et al., 2021; Lages et al., 2021). Recently, *Campylobacter* species, especially *C*. *coli* and *C*. *jejuni*, have been recognized as the most prevalent pathogens associated with chicken and meat products in both developed and developing countries, causing campylobacteriosis in humans (Guirin et al., 2020). After *Campylobacter* spp., the second most important bacterial pathogen is *Salmonella* spp. isolated from various foodstuffs causing vomiting, diarrhea, fever, abdominal cramps, headache, and blood in feces. According to the World Health Organization (WHO), in the United States of America, *Salmonella typhi* was responsible for the infection of 750,000 people, leading to more than 52,000 deaths annually (World Health Organization [WHO], 2015). *Listeria monocytogenes* is another major food contaminating pathogen causing listeriosis (Välimaa et al., 2015). Risks of listeriosis are commonly observed in pregnant women, infants, and immunocompromised patients, while healthy people showed mild symptoms (Gholipour et al., 2020). *Escherichia coli* is another important food contaminating pathogen associated with beef and beef products secreting the Shigha toxin (Shigha toxin-producing strain of *E*. *coli*- STEC) eventually causing bloody diarrhea and low platelet counts in affected patients (Niveditha et al., 2021).

Apart from bacterial contamination, several fungal species *viz*. *Aspergillus*, *Fusarium*, *Penicillium*, *Alternaria*, *Cladosporium*, *Rizopus*, and *Mucur* are also registered to be responsible for spoilage of fresh fruits, vegetables, stored grains, meats, and other essential food products (Axel et al., 2017; Hu et al., 2019; Atif et al., 2020; Das S. et al., 2020). In addition to food spoilage, fungal pathogens also deteriorate food products by producing health-hazardous toxic secondary metabolites, called mycotoxins (Alizadeh et al., 2020). Moreover, frequent contamination of preharvest and postharvest stored food products by *Aspergillus*, *Penicillium*, and *Fusarium* secretes more than one mycotoxin leading to significant economic losses (Chaudhari et al., 2020). Among all 400 reported mycotoxins, aflatoxin, zearelenone, fumonisins, ochratoxins, and deoxynivalenol are major health-hazardous mycotoxins posing adverse effects on mammalian health after consumption of contaminated foods (Aldars-Garcia et al., 2016).

Foodborne pathogens deteriorate the food quality, reduce the content of important nutrients and vitamins, and shorten the shelf life of food products by releasing extracellular enzymes and changing the odor, texture, and overall appearance of foodstuffs (Deepika et al., 2021). Spoilage of food products is rarely investigated unless it has taken the form of an outbreak. Thus, the microbial spoilage and wastage of food and food products demands extensive research in the area of natural products including the application of EOs with an objective to secure public health through long-term preservation of different food commodities.

## Nanoencapsulated Essential Oils for Food Preservation

Essential oils are volatile secondary metabolites obtained from different parts of medicinal and aromatic plants such as the leaves, stem, flowers, fruits, and buds (Falleh et al., 2019). Nearly, 3000 EOs have been isolated from 2000 plant species, and of them, 300 EOs are known to be utilized for commercial purposes. EOs are complex mixture of several bioactive components such as terpenes, terpenoids, alcohols, esters, ketones, phenylpropanoids, and aldehydes. Many synthetic preservatives have been used against microbial contamination in stored food items, but these preservatives have several adverse effects on human health and the environment. Therefore, current researchers are focused toward plant-based preservatives having negligible side effects.

In line with the non-toxic nature of EOs, some of them have been used as food preservatives. For instance, Eco-SMART is one of the most popular EOs based preservatives used in industry. Eugenol Tween^®^ and Eugenol ethoxylate, Pycnogenol^®^ and Hebalox^®^, DMC Base Natural, and Protecta One and Protecta Two are EO-based preservatives used for the protection of harmful pathogens commonly encountered in food items (Prakash and Kiran, 2016).

The current studies in food safety have witnessed the success of EOs in controlling food spoilage by Gram-positive and Gram-negative bacteria, fungi, and the toxins secreted by them. However, the wide-scale application of EOs as free form in food is limited due to:

(a) Rapid release from applied surfaces,
(b) Possibility in the changes of food organoleptic properties caused by intense aroma,
(c) Oxidation of EO components by environmental factors like temperature, irradiation, and moisture, and
(d) Considerable loss in EO biological activity.

Nanoencapsulation has emerged as an important technique to entrap EOs and bioactive compounds with an objective to improve the microbial inhibitory activities, antioxidant properties, and utilization in real food systems (Dwivedy et al., 2018; Chaudhari et al., 2021a). Nanoencapsulation encompasses the natural products or compounds of interest in a compatible polymeric matrix with a minimum of one dimension below 100 nanometers (Prakash et al., 2018). Loss in the availability of free EO at applied food surfaces after short durations restricts their potentiality in the food system. Controlled release, therefore, could be of considerable interest to improve the longevity of shelved food products. Controlled release of EOs loaded on zein nanoparticles has been presented to preserve meat (Xavier et al., 2021). The authors also demonstrated preserved antioxidant activity of *Cinnamodendron dinisii* EO after incorporation in a chitosan matrix. Mahdi et al. (2021) reported an enhancement in the oxidative stability of *Citrus reticulata* EO after encapsulation in composite wall materials comprising of whey protein, maltodextrin, and gum arabic. In addition, nanoencapsulated EOs are registered to exhibit enhanced antioxidant activity and solubility, as well as stability against increased temperature (Hadidi et al., 2021; Yang et al., 2021). Since the strong flavor and aroma of some EOs may modify the sensorial attributes of treated food, nanoencapsulation may be performed to facilitate the controlled release and hence the preservation of sensory properties. The positive influence of nanoformulated *Cinnamomum zeylanicum* EO in comparison to free EO on sensory attributes of beef patties has been demonstrated by Ghaderi-Ghahfarokhi et al. (2017). The patties color was more stable after EO encapsulation, suggesting the effectiveness of fabricated formulation in improving the organoleptic attributes of treated meat. Therefore, nanoencapsulation is a promising approach to augment stability, distribution, and delivery of EOs and visible characteristics of treated foods.

## Methods of Encapsulation of Essential Oils (Fabrication of Nanoemulsion)

Fabrication of nanoemulsion can be classified into:

(i) Methods involving nanoemulsion synthesis, and
(ii) Ingredients/major components of nanoemulsion.

### Methods for Fabrication of Nanoemulsion

Nanoencapsulation of EO into a suitable polymer matrix in the form of nanoemulsion has been performed by different processes *viz*. nanoprecipitation, inclusion complexation, solvent evaporation-emulsification, coacervation, and supercritical fluid.

Nanoprecipitation is a common technique for the encompassment of lipophilic components like EOs into semipolar solvents and polymers through interfacial deposition. The method is easy, reproducible, and commonly used due to the its minimal energy use and simplicity (Eisner, 2014). Commonly used polymers for encapsulation of lipiophilic components involve poly (lactic-co-glycolic acid), poly (alkylcyanoacrylate) (PACA), polylactic acid (PLA), and poly(ε-caprolactone). More importantly, for improvement in functionalities of core material, controlled delivery, and cellular uptake, multiple biopolymer matrices have been used (Walia et al., 2019).

Inclusion complexation is another important method of encapsulation involving molecular linkage between the core material and matrix polymer. Molecular linkage during the encapsulation process includes Van der Waal forces, hydrogen bonding, and hydrophobic interactions with a high yield of nanoparticles (Aree and Jongrungruangchok, 2016). In this method, β-cyclodextrin and β-lactoglobin have especially been recognized as suitable nanocarriers for encapsulation of lipophilic components (Walia et al., 2019).

Solvent evaporation-emulsification involves polymer solution emulsification followed by solvent evaporation and subsequent formation of nanoparticles. Ethyl cellulose, polycaprolactone, polylactic-co-glycolic acid (PLGA), and polylactic acid (PLA) are commonly used polymers for the development of nanospheres by employing high-speed homogenization and ultrasonication (Fornaguera et al., 2015). Tsai et al. (2011, 2012) reported high-pressure emulsification for effective incorporation of curcumin in PLGA with improved bioavailability.

The coacervation process allows differentiation and phase separation of a single polymer matrix or a mixture of polymer matrices followed by encircling of the core phase. Cross-linking in hydrocolloid shells has occurred in presence of enzymatic and chemical cross-linkers such as transglutaminase and glutaraldehyde that help to increase the coacervate robustness (Ezhilarasi et al., 2013). On the basis of the number of polymers used, the process has been differentiated into simple and complex coacervation. More importantly, encircling/coacervate strength depends on chemical/enzymatic cross-linkers, ionic strength, pH, biopolymer type, concentration, and the nature of the complex formed (de Kruif et al., 2004). Hu et al. (2018) synthesized cinnamon-thyme-ginger composite essential oil nanocapsule by complex coacervation with the involvement of chitosan as biopolymer and tripolyphosphate as cross-linking agent.

A supercritical fluid is used for encapsulation of thermally sensitive bioactive compounds, followed by evaporation of fluid by spraying process and further precipitation of the solute particles. Arango-Ruiz et al. (2018) reported encapsulation of curcumin into polyvinylpyrrolidone by supercritical antisolvent technology. In an investigation by Türk and Lietzow (2004), nanoencapsulation of phytosterol was performed by supercritical fluid with particle size less than 500 nm.

### Ingredients and Components of Nanoemulsion

Major ingredients of nanoemulsion are EO, surfactant, and water (Dasgupta et al., 2019). Proper mixing of these components regulates the properties and stability of the emulsion. The selection of surfactants during nanoemulsion formation is dependent on the surface’s active nature emphasizing the stability, pH, temperature, and ionic strength of the nanoemulsion system (McClements et al., 2017). Moreover, the water-to-oil ratio determines the stability and size of particles in nanoemulsion systems. In addition to EO, surfactant and water, thickening agent, weighting agent, emulsification, antioxidants, and polyunsaturated fats also improve the dispersion stability of nanoemulsion (Dasgupta and Ranjan, 2018). The amount of water, as well as its unique properties, greatly influence the organoleptic property of foods. Water crystals in emulsion have a significant effect on the texture and taste of food products. Emulsifier also helps in the prevention of coalescence and flocculation in nanoemulsion by interfacial interaction. Emulsifier facilitates in droplet break up leading to the formation of small size particles. The concentration of emulsifier is decided on the basis of the amount of biopolymer to cover all the oil- water interfaces and the rate of coating. EOs are used as oil phase and have more tendency to protect themselves from oxidative degradation after encapsulation into biopolymer in nanoemulsion system (Dasgupta et al., 2016a).

## Role of Essential Oils Nanoemulsion as Food Additives

Recently, consumers as well as modern food industries are focusing on nanoengineered EOs in the forms of nanoemulsion to avoid the drawbacks of unencapsulated EOs for practical utilization with maximum stability and compatibility. Further, nanoemulsions provide maximum benefits associated with the use of EOs in food items such as:

(a) Increased dispersion within the food surfaces where microbes generally multiply.
(b) Reduced sensorial effects.
(c) Increased antimicrobial activity of nanoemulsions containing bioactive molecules of EO (Donsì and Ferrari, 2016).
(d) Due to multiple targets sites in microbial cells, the emulsion-based delivery systems containing EOs may easily interact with microbes, thus interfering with the normal biological activities.
(e) Most importantly, different components present in EOs either individually or in combination with other components may exhibit synergism and conceivably play a crucial effect in membrane disruption, leakage of cytoplasmic constituents, and metabolic alterations.

### Antibacterial Efficacy of Essential Oil Nanoemulsion

The antibacterial efficacy of EOs is a research hotspot to fulfill the need for natural antibacterial compounds. However, in most of the studies, EOs showed higher activity against Gram-positive than Gram-negative bacteria. This mainly happens because of structural differences in the membrane of Gram-positive and Gram-negative bacteria in terms of the association of hydrophobic compounds (Franklyne et al., 2016). A number of researchers have added valuable information about the antibacterial activity of EO nanoemulsions against a wide range of food contaminating bacteria.

#### Efficacy of Essential Oil Nanoemulsion Against *Listeria monocytogenes*

*Listeria monocytogenes* is the major bacteria responsible for foodborne illnesses in the food sector, especially ready-to-eat foods causing disease outbreaks mainly in the US and in other parts of the world (Harris et al., 2003). *L. monocytogenes* is the main causal agent of listeriosis, a very common foodborne illness. In this context, nanoencapsulated EOs are an effective agent and have been substantially used to control *L*. *monocytogenes* growth in vegetables, fruits, and ready-to-eat foods. Bhargava et al. (2015) artificially inoculated fresh lettuce with *L. monocytogenes* and dipped it in *Origanum* spp. oil nanoemulsions for 1 min. At 0.1% concentration, they observed up to 3.57 log CFU/g reduction that was further confirmed by disruption in the cell membrane as revealed through SEM observation. Paudel et al. (2019) in a similar way artificially inoculated honeydew and cantaloupe with *L. monocytogenes* followed by treatment with cinnamon oil nanoemulsions for one minute. The treatment showed up to 7.7 log reductions in bacterial growth at 0.5% nanoemulsion. In another study, Maté et al. (2016) reported the combined effect of d-Limonene nanoemulsion with heat stress for inhibition of *L. monocytogenes*. The thermal resistance of *L*. *monocytogenes* was reduced two to five times when 0.5 mM D-limonene was added to the heating medium. Moreover, when the same concentration of D-limonene nanoemulsion was added to the heating medium, the resistance was reduced by more than one hundred times and showed very promising results on the inactivation of microorganisms by the combined effect of nanoemulsified D-limonene and thermal treatments. These studies suggested that EO-based nanoemulsions can be used as effective natural antibacterial agents against *L. monocytogenes* contamination in the food industry.

#### Efficacy of Essential Oils Nanoemulsion Against *Salmonella* Species

In a study, Moghimi et al. (2016a) tested Sage (*Salvia officinalis*) EO nanoemulsion against *S. typhi* and found four times higher activity than the unencapsulated EO. The nanoemulsion treatment showed extensive cell membrane damage as determined by efflux of cellular protein, DNA/RNA, and Mg^2+^, K^+^, and Ca^2+^ in the extracellular media during *in vitro* testing. In another study, Kang and Song (2018) spot-inoculated red mustard leaves with *Salmonella typhimurium* and treated them with nanoemulsions containing different EOs. The investigation observed higher reductions as compared to 0.02% NaOCl. More importantly, the nanoemulsion significantly maintained the sensory properties of red mustard along with nutritional qualities. Hadian et al. (2017) demonstrated the antibacterial activity of nanogel encapsulated *Rosmarinus officinalis* EO (REO) against *S. typhimurium* on beef cutlet samples. They reported that encapsulated REOs coating on beef cutlets were more effective as compared to free REOs in controlling the *Salmonella* population under refrigerated storage. Nanoencapsulation effectively reduced the microbial population by 2 mg/g beef cutlet.

#### Efficacy of Essential Oil Nanoemulsion Against *Escherichia coli*

He et al. (2021) observed the stronger microbial inhibitory activity of thyme (*Thymus daenensis*) EO nanoemulsion as compared to the EO alone or coarse emulsion against *E. coli* O157:H7. Further, the combination of the synergistic actions of ultrasound (US) and EO nanoemulsion showed remarkable decontamination of *E. coli* on contaminated cherry tomatoes without affecting firmness and color. Similarly, Guo et al. (2020) observed the morphological changes in *E. coli* cells after treatment as observed through SEM, TEM, and Laser scanning confocal microscopy. The synergistic effects of the ultrasound and oil nanoemulsion caused changes in the morphology, interior microstructure of cells, and permeability of cell membranes leading to increased release of nucleic acids and proteins. The study provided valuable information with reference to the potential of EO nanoemulsions in food preservation. However, in a similar study, Salvia-Trujillo et al. (2014) proposed that microfluidization rather than ultrasounds seemed to have an improved antimicrobial activity of *Cymbopogon citratus* EO against *E. coli*. Moraes-Lovison et al. (2017) evaluated the antibacterial and physiochemical stability of nanoencapsulated *Origanum vulgare* EO. In this investigation, *in vitro* minimum inhibitory concentration (MIC) and minimum bactericidal concentration (MBC) against *E. coli* at were recorded as 0.60 and 3.32 mg/mL, respectively. They also observed the physiochemical characteristics of meat products by incorporation of nanoemulsion in chicken pâté and found negligible changes in meat products. Moghimi et al. (2016b) developed high-intensity ultrasound-based water-dispersible *Thymus daenensis* EO nanoemulsion (diameter = 143 nm). The EO nanoemulsion showed a high inhibitory efficacy against *E*. *coli* with MIC at 0.4 mg/mL. The nanoemulsion exhibited 10 times more antibacterial activity than the free EO, thus the conversion of EO into nano-scale particles greatly enhanced the bactericidal activity.

#### Efficacy of Essential Oil Nanoemulsion Against *Bacillus* Species

Although many *Bacillus* spp. are non-pathogenic, they can exhibit hemolysis and are raising concern because of their extensive use as a model organism. Several case studies have confirmed severe health impacts associated with the consumption of *Bacillus* spp. contaminated food (Singh N. et al., 2020). There are many reports related with the *in vitro* studies of EO nanoemulsions against different *Bacillus* spp. Hassanshahian et al. (2020) observed MIC and MBC values of *Alhagi maurorum* EO nanoemulsion as 1.75 and 6.25 mg/ml, respectively against *B. cereus* and found it two times more effective than that of the EO alone using the macro-broth dilution method. Ghosh et al. (2013) observed a significant reduction in *B. cereus* population even at a higher dilution of *Cinnamomum zeylanicum* EO nanoemulsion. They also demonstrated membrane distortion in treated cells as observed through vital cellular constituents leakage, EtBr staining, and SEM analysis. In a similar study, Zhang et al. (2017) observed that encapsulated cloves/cinnamon EO nanoemulsions displayed prominent antimicrobial effectiveness against *B. subtilis* as compared to the non-nanoemulsion counterparts. Further, the addition of EO nanoemulsion in a sauce prepared from mushrooms did not alter the flavor, offering practical applicability for food preservation. Frank et al. (2018) evaluated the antibacterial activity of Cinnamon EO nanoemulsion (CEO-NE) incorporated into alginate against *B. cereus*. Thus, they reported increased inhibition of *Bacillus* by enhancing the concentration of CEO-NE from 20–40%, respectively.

Supplementary Table 1 presents the antibacterial efficacy of EOs nanoemulsion focusing on the type of EO with their active constituents, microbes categorization, and effective mechanisms in foods.

### Antifungal Activity of Essential Oils Nanoemulsion

As most of the studies dealing with antimicrobial activity of EO nanoemulsions have focused on bacteria, the study with respect to antifungal activity is sparse. However, some valuable information on the antifungal activity of EO nanoemulsions against some food-borne fungal strains has been added by some researchers during the last decades. The food items are most commonly contaminated with *Aspergillus*, *Fusarium*, and *Penicillium* causing severe health hazards to consumers, oxidative deterioration, and lipid peroxidation of contaminated food due to their mycotoxin-producing ability (Ribeiro et al., 2019).

#### Efficacy of Essential Oil Nanoemulsion Against *Aspergillus* Species

Among different mycotoxins, aflatoxins secreted by *Aspergillus* are the most common and serious food contaminant. During *in vitro* studies, Das S. et al. (2020) observed better fungitoxic efficacy of *Myristica fragrans* EO nanoemulsion (1.75 μl/ml) than the EO alone (2.75 μl/ml) against a toxigenic strain of *A*. *flavus*. A similar finding was also observed when tested *in vivo* in rice food samples stored for 6 months. The authors further suggested that the superior activity of nanoemulsion was due to the subcellular size particles with targeted delivery of components. In another study, Ribes et al. (2017) observed enhanced inhibitory effect of *Cinnamomum* spp. leaf EO nanoemulsions than free EO against *A. niger* mycelial growth and spore germination. The authors further suggested that the antifungal action was correlated with the loss of cytoplasm in hyphae and hyphal tip as observed in *A. niger*. Another interesting finding of the work has been associated with the implication of nanoemulsions for the preparation of effective and stable natural antifungal agents in food-based applications.

#### Efficacy of Essential Oil Nanoemulsion Against *Penicillium* Species

Mahajan et al. (2021) tested *Ocimum gratissimum* EO and its nanoemulsions against *P. digitatum* of kinnow mandarin fruit by the poisoned food technique. The nanoemulsion exhibited stronger growth inhibition (1 × 10^4^ CFU ml^–1^, 96%) than unencapsulated oil (13 × 10^4^ CFU ml^–1^, 85%) on the 15^th^ day of incubation. Further, the SEM and optical microscopy suggested stronger suppressive activity of EO nanoemulsions for germination of spore and elongation of hyphae in *P. digitatum*. In a similar study of interest, Long et al. (2020) observed about 300 times more bioactivity as MIC value changed from 3.7% to 0.013% when garlic oil nanoemulsion was tested in comparison to garlic oil alone against *P. italicum*, a common contaminant causing postharvest decay of fruits and vegetables. The antifungal mode of action of oil nanoemulsion showed malformation in cell structure with destroyed lipids, nucleic acids, and proteins. Further, the oil nanoemulsion also successfully inhibited *P. italicum* infestation in citrus during *in vivo* trials, thereby strengthening its use as a suitable alternative to fungal contamination in fruits and vegetables.

#### Efficacy of Essential Oil Nanoemulsion Against *Fusarium* Species

Wan et al. (2019) tested fungitoxic potentiality of lemongrass, clove, peppermint, thyme, and cinnamon EO nanoemulsions against *F. graminearum* using the agar dilution method. The thyme oil nanoemulsion showed the strongest antifungal activity (EC_90_ = 7.25–7.61 mg/g), while the peppermint oil nanoemulsion showed the lowest (EC_90_ = 23.67–23.84 mg/g) activity against mycelial growth of both strains. The authors also suggested that the strong activity of thyme EO nanoemulsion was due to the presence of phenol, i.e., thymol causing disruption in ergosterol biosynthesis and membrane integrity. In another study, Abd-Elsalam and Khokhlov (2015) observed suppression of mycelial proliferation in *F. oxysporum* f. sp. vasinfectum, the causal agent of wilt of cotton by eugenol loaded nanoemulsion.

#### Efficacy of Essential Oils Nanoemulsion Against *Rhizopus* spp.

*Rhizopus* is the main causal agent of soft rot in fruits and vegetables during storage. In a study, Yousef et al. (2019) observed MIC value 1000 μl/L of cinnamon EO nanoemulsion in potato dextrose agar medium against *R. stolonifera*, causing rot of strawberry. In general, the EO nanoemulsion showed stronger antifungal activity than the EO alone and the common synthetic fungicide thiabendazole. In addition, the EO nanoemulsion also caused significant fruit decay reduction and the lowest fruit infection (5.43%) at 0.2% concentration. In a similar study, cinnamon EO nanoemulsion also showed better efficacy than the EO coarse emulsion and a common antifungal drug Amphotericin B against *R. arrhizus* when examined through the disk diffusion method (Pongsumpun et al., 2020). The authors suggested better efficacy of nanoemulsion facilitated by small sizes particles delivering EOs to the fungal cell membrane, while the coarse emulsion was due to low solubility in water, and could not interact with the cell membrane properly. These studies proved the efficacy of cinnamon EO nanoemulsion as natural fungicides for the control of postharvest losses caused by *Rhizopus* spp. A list of important studies indicating the inhibitory potential of EOs nanoemulsion against different storage fungi along with the key findings is presented in Supplementary Table 2.

## Antimicrobial Mechanism of Action of Essential Oils Nanoemulsion

Essential oils recognized as secondary metabolites have been obtained from aromatic plant families such as *Lamiaceae*, *Asteraceae*, *Myrtaceae*, *Apiaceae*, *Rutaceae*, *Zingiberaceae*, and many others. Naturally, EOs are lipophilic or hydrophilic in nature and are complex mixtures of hundreds of unstable and non-volatile active organic compounds and may be characterized into terpene (e.g., limonene and myrcene), terpenoids (linalool and Thymol), and phenylpropanoids (anethole, eugenol) (Hassoun and Çoban, 2017; Pateiro et al., 2021). Major bioactive components responsible for antimicrobial activity in various EOs include the presence of phenolic components such as carvacrol, eugenol, and thymol in EOs which exhibit strong antimicrobial properties, followed by terpenes and ketones (Li et al., 2015; Pathania et al., 2018). In addition to major components, minor components of EOs also play a significant role against microbes owing to synergistic activity between minor and major components (Bhavaniramya et al., 2019). However, the mechanism of action of EOs and their components is largely restricted due to low solubility, poor bioavailability, and quick release. The drawbacks associated with EOs alone could be overcome by encapsulation. Therefore, nanoencapsulated EOs having enhanced antimicrobial activity can be used in various food sectors against different food spoilage microbes.

**FIGURE 1:**
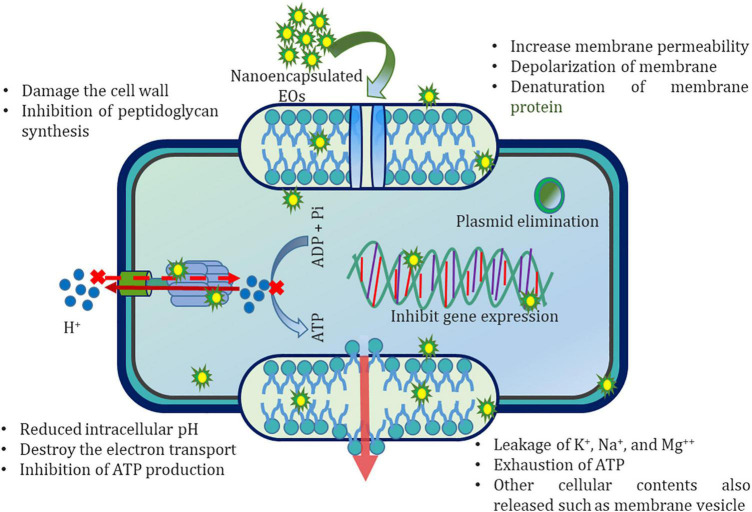
Schematic representation of possible antibacterial mechanisms of action of EO nanoemulsion.

Benjemaa et al. (2018) reported that the antibacterial activity of EOs was enhanced and prolonged after nanoencapsulation. Usually, the antibacterial activity of nanoencapsulated EOs cannot be assigned to a single mechanism of action due to the presence of different bioactive components of EOs having multiple functional groups in their chemical composition (Diao et al., 2014), facilitating different routes for their possible action on microbial cells. Many researchers have presumed an enhancement in the specific mechanism of action of nanoencapsulated EOs as compared to free EOs (Moghimi et al., 2016b; Lu et al., 2018; Chu et al., 2020; El-Sayed and El-Sayed, 2021). In this reference, nanoencapsulated EOs provoked the disorganization of the phospholipid bilayer of bacterial cell membrane and mitochondria, damage to membrane proteins, followed by the increase in cellular permeability, instability of cellular structure, and the depletion of proton motive force, electron flow, and active transport (Benjemaa et al., 2018; Bhavaniramya et al., 2019; Yang et al., 2021). Consequently, the presence of EO enhances the leakage of vital cellular ions (Na^+^, Mg^2+^, K^+^) and 260 and 280 nm absorbing cellular constituents such as DNA, RNA, and proteins (Figure 1), leading to significant changes in the bacterial cell responsible for cell death.

**FIGURE 2:**
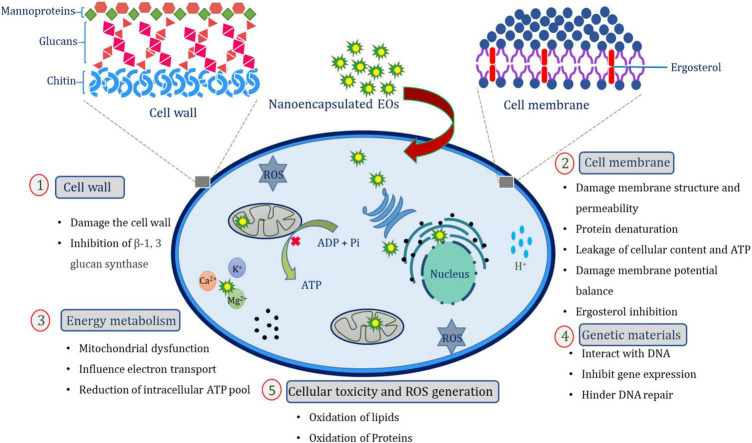
Schematic representation of possible antifungal mechanisms of action of EO nanoemulsion.

Notably, nanoencapsulated EOs have also been shown to exhibit strong activity against food-borne fungal pathogens. Due to their complex mixture of bioactive components, the exact antifungal mechanism of action is not fully unveiled. A possible antifungal mechanism of action of nanoencapsulated EOs is schematically presented in Figure 2. Several studies have indicated that the fungitoxic efficacy of nanoencapsulated EOs and their active constituents are the outcome of interferences in the biosynthesis of the cell wall rendered by the larger surface area of nanoparticles and modulation in ionic permeability of the fungal plasma membrane. Primarily, nanoencapsulated EOs target cell membrane due to the lipophilic nature of EOs allowing mobilization across the fungal cell membrane leading to contraction in the partitioning of the lipid bilayer, damage of cellular integrity and alteration in membrane permeability, leakage of vital intracellular components (Ca^2+^, K^+^, and Mg^2+^), inhibition of mitochondrial electron transport system, reduction in the membrane potential by inhibiting the proton pump with subsequent loss in the ATP pool, and eventually apoptosis (Kalagatur et al., 2018; Singh A. et al., 2020). More importantly, ergosterol is maximally affected by EO nanoemulsions leading to destabilization of membrane integrity and stability (Chaudhari et al., 2021a). An investigation conducted by Singh et al. (2019) and Li et al. (2020), revealed that *Ocimum sanctum* and *Illicium verum* EOs, respectively can induce considerable impairment in ergosterol biosynthesis in *Aspergillus flavus.* Thus, nanoencapsulated EOs can be utilized as possible natural antimicrobial agents against food spoilage pathogens.

## Application of Nanoemulsion

Nanoemulsions have various applications for encapsulation, protection, and delivery of bioactive components such as nutraceuticals (food components having health benefits), pharmaceuticals (drugs), flavor improvement, and antioxidant qualities in foods.

Nanoemulsions containing lipophilic functional compounds *viz*. flavonoids, phytosterol, carotenoids, and fat-soluble vitamins have multiple applications in biomedical and health aspects. Guttoff et al. (2015) synthesized vitamin D nanoemulsion by spontaneous emulsification and studied the effect of composition and preparatory conditions on emulsion stability. Meghani et al. (2018) reported the potentiality of vitamin D encapsulated cinnamon essential oil nanoemulsion to arrest the cell cycle progression, increased caspase activity, and decreased expression of BcL2 protein leading to loss of mitochondrial membrane potential in human alveolar carcinoma cells. Encapsulations of bioactive components of *Tinospora cordifolia* extract *viz*. berberine, palmatine, and palmatoside into whey protein through electrospraying nanospheres with controlled delivery for anti-diabetic activity have been recently investigated by Jain et al. (2021). The synthesis of silver nanoparticles with the involvement of black cumin seed extract and its affectivity as an antidiabetic and anti-inflammatory agent has been investigated by Vijayakumar et al. (2021a, b). Agarwal et al. (2021) developed curcumin-loaded polycaprolactone/polyvinyl alcohol silk-fibroin based electrospun nanofibrous mat for improved antidiabetic activity with controlled delivery. In addition to nanoemulsion, metal nanoparticles also have a prime role in biomedical applications such as drug delivery, imaging, gene delivery, bio-labeling, and tissue engineering. Vijayakumar et al. (2021b) synthesized zinc oxide nanoparticles by involving neem gum as a capping agent and analyzed the activity for inhibition of cell cycle proliferation in Hep G2 human cancer cell line.

Nanoemulsions have extended their applicability for improvement in air dispersion and foam stability in sugar and flour confectionery products. The non-fat particles of chocolate such as cocoa, milk, and sugar are suspended in cocoa butter (fat phase). Moreover, in the nanoemulsion form, emulsifiers help in the prevention of blooming, a major cause of sensorial alteration. The cream of bakery products in sandwiches and cakes poses huge market demands in the form of emulsions. Nanoemulsion has been used in ice cream for effective improvement in texture and uniformity (Prasad et al., 2017).

Nanotechnology has more promising applications in the field of agriculture, food preservation, and biomedicals in the form of nanosensors. In addition to EO-loaded nanoparticles, copper, silica, and zinc nanoparticles showed effective potentiality for disease management, systemic acquired resistance, and also act as novel antimicrobial agents for the management of pathogens affecting agricultural crops, animals, and humans (Chhipa and Joshi, 2016; Gandhi et al., 2021). Plant-based nanoemulsions in conjunction with polymers develop smart nanosensors that can be used in food packaging and detection of agricultural food quality. Most notably, two different types of nanosensors *viz*. phytonanosensor and electrical nanosensors are being used in agriculture. Copper nanoparticles with nanogold electrodes have been used to detect salicylic acid levels in oilseed rape infected by fungal pathogen *Sclerotinia sclerotiorum* (Wang et al., 2010). Phyto/bionanosensor in combination with nanoparticles interpreted the quality of food by color changes without any laboratory testing. Owing to high toxicity, solubility, and indiscriminate utilization of pesticides in the agriculture and food industries, there is an urgent need for nanosensor technology for residue analysis of these pollutants (Valdés et al., 2009). Moreover, nanoparticle-based nanosensors can act as a smart delivery vehicle to protect agricultural crops from pathogens and facilitate an improvement in agrochemicals in low proportions (Thiruvengadam et al., 2018).

Practical application of EO nanoemulsion in food products as an antimicrobial agent is a challenging task. Nanoemulsions containing EO display a greater surface-to-volume ratio and more easily controlled delivery, with an improvement in their antimicrobial properties. In particular, the food-based application of nanoemulsions has been classified into different frontier areas:

(a) Direct mixing with food products,
(b) Infusion in porous food matrices,
(c) Food surface washing with antimicrobial nanoemulsion, and
(d) Coating of food products by nanoemulsion.

The application of nanoencapsulated EOs in the active packaging of food products is presented in Supplementary Table 3.

In a recent investigation by Singh P. et al. (2020), it has been observed that clove oil nanoemulsion effectively reduced the growth of *Fusarium proliferatum* and secretion of fumonisin B_1_ and B_2_ in maize kernels during storage conditions. More importantly, nanoemulsion not only enabled controlled delivery of EO or antimicrobial compounds, but also facilitated incorporation into complex food systems. Dasgupta et al. (2016a) reported food grade vitamin E acetate nanoemulsion using edible mustard oil with improved antimicrobial and antioxidant efficacy. Strong antibacterial and antibiofilm impact of nano-silver decorated *Ocimum basilicum* leaf extract has been reported by Muthulakshmi et al. (2021). Corresponding to complex mechanism, nanoemulsion enhanced the passive cellular absorption, reduced the mass transfer resistance, and as a result increased the antimicrobial activity (Ranjan et al., 2016). Additionally, nanotechnology has a prime role in nanofood packaging, particularly in smart and active packaging with resultant inhibition of microbial infection (Singh et al., 2018).

## Safety Profile of Essential Oils and Nanoemulsion

In addition to microbial infestation and toxin inhibitory efficacy, large scale practical recommendation of EOs and nanoemulsions as an effective food preservative require safety assessment without any toxic effects on mammals make them healthier for consumers. To address this issue, different international organizations such as the Food Chemical Codex, International Organization of Flavor Industries, Food and Drug Administration, Codex Alimentarium and the Council of Europe, and the Flavor and Extract Manufacturers Association have confirmed specific procedures for toxicological and chemical characterization of EOs. They also reported antagonistic and synergistic effects for specific EOs in mammals during practical application (Falleh et al., 2020; Chaudhari et al., 2021b). Generally, mammalian safety/toxicity of EOs and nanoemulsions were performed on experimental mice/rats which permits the determination of safety assessment in terms of Median Lethal Dose or LD_50_ value. In this context, Deepika et al. (2021) performed acute toxicity assay of *Petroselinum crispum* essential oil (PEO) and chitosan encapsulated PEO in mice and the LD_50_ value was found to be 10,765 and 26,830 mg/kg body weight, respectively. Similarly, Das et al. (2021b) in a study assessed the acute oral toxicity of *Pimpinella anisum* essential oil (PAEO) and chitosan nanostructured PAEO in mice, and LD_50_ were displayed as 19,879.89 and 13,641.35 μl/kg body weight, respectively. They suggested that the lower LD_50_ value of nanostructured PAEO might be due to a small-sized nanoemulsion with more EO in each nanocapsule and reduced the Median Lethal Dose. In another research, Dwivedy et al. (2018) reported the acute toxicity of *Illicium verum* essential oil (IvEO) on male mice in terms of Median Lethal Dose and found it to be 11,257.14 μl/kg body weight. EOs and nanoemulsions having higher LD_50_ values as compared to other synthetic food preservatives like bavistin (1500 mg/kg), nystatin (8000 mg/kg), and lindane (59-562 mg/kg) strengthen their application in food and agricultural industries as eco-friendly and safe preservatives.

In spite of proven safety in different model organisms, toxicities have also been reported. Synthesis of nanoemulsionic particles of tin oxide by using *Piper nigrum* seed extract and toxic effects on cancer cell lines has been investigated by Tammina et al. (2017). More importantly, the toxicity of nanoparticles is very complex and depends on different physico-chemical properties such as shape, size, charge, and reactivity (Fadeel and Garcia-Bennett, 2010). Direct ingestion of nanomaterials may occur through drug delivery and food packaging-based applications. Nanomaterials after ingestion have been translocated to the intestinal lumen by blood circulation (Pietroiusti et al., 2013). In addition to size, the shape of nanomaterials also determines the toxicity, for example triangular-shaped nanoparticles are more toxic as compared to spherical nanoparticles (Dasgupta et al., 2016b). There are a number of risks that have been associated with the use of nanoparticles which have been focused by different regulatory agencies. The Royal Society and Royal Academy of Engineering in the UK significantly anticipate and regulate health-related problems associated with nanoparticles. The European Commission’s Scientific Committee on Emerging and Newly Identified Health Risks has also realized the nanoparticle toxicity problems in human health as well as the environment (Sharma et al., 2012). The European Commission’s Scientific Committee on Consumer Products (SCCP) suggested the inappropriateness of excessive nanoparticle utilization and that there was inadequate information of uptake and absorption of nanoparticles by the skin and other cell organs. Mosa et al. (2018) reported that the toxicity of nanoparticles has been associated with liver complications and severe ill health. Most notably, nanoparticles by virtue of their small size, induce the chances of genotoxicity by directly interacting with DNA/RNA or causing indirect damage by ROS (Ranjan et al., 2019). Nanoparticles also interacte with nuclear and cytoplasmic proteins leading to interruption of antioxidant defense mechanisms (Jain et al., 2018). Therefore, there is a need for comprehensive investigation of nanoparticle uptake, entry into the food chain, and distribution both under *in vitro* and *in vivo* conditions to extend the applicability of EOs based nanoemulsion in food safety.

## Market Demand of Nanoemulsion Based Nutraceuticals and Pharmaceuticals

The application of nanoemulsion in nutraceuticals, pharmaceuticals, and food products has huge market demands along with consumer preferences. Nanoemulsion basically contributes a lower optical transparency than the wavelength of light in the production of beverages and foods (Dasgupta et al., 2019). Furthermore, nanoemulsion also facilitates in the expansion of the functional food market by incorporating lipophilic bioactive components. The field of nutraceuticals is considering the advantages of the incorporation of innovative nanotechnology with controlled delivery from nano-nutraceuticals, nanoemulsion, and more importantly liposome-based delivery. Moreover, nanoemulsion-based delivery improves bioavailability and fulfills the gap between active substance content and bioaccessibility (Daliu et al., 2019). Aquanova, a commercial nanotech industry developed a number of nanocarrier systems with the encapsulation of vitamin E, vitamin C, and fatty acids for pharmaceutical and nutraceutical applications. Zyme and Aquanova synthesized omega-3 fatty acid nanocapsules as a commercial product with high market demand. NutraLease, a similar company, like Aquanova developed a nanoemulsion containing various functional compounds like lycopenes, isoflavones, vitamins (A, D3, and E), and phytosterol and have been found stable at various stages of processing (Silva et al., 2012; Ranjan et al., 2014; Dasgupta et al., 2015). Nano-self-assembled structured liquids (NSSLs), a flavor encapsulating nanoemulsion developed by NutraLease is reported to the enhance bioaccessibility and bioavailability of nutraceuticals (Jaiswal et al., 2015).

## Concluding Remarks and Future Research Opportunities

Microbial food spoilage caused by bacterial and fungal contamination has documented the rising cases of diseases outbreaks and massive human deaths globally. Although the employment of synthetic chemicals to control microbial food deterioration has received considerable success, the toxicity to human health and the environment, induction of resistance development, and the presence of residues in treated food samples have necessitated the search for preservatives of natural origin. Among natural products, EOs derived from aromatic plants have received considerable attention from the food industry because of their safety, negligible chances of residual toxicity, considerable antimicrobial activity, and promising antioxidant activity. Nevertheless, poor water solubility, high volatility, and the intense aroma of EOs have restricted their application in the food system. The limitations associated with EO could be resolved by encapsulation in suitable polymeric matrices including chitosan, alginate, zein, carrageenan, polycaprolactone, and cyclodextrins. Various studies have reported the improved antimicrobial of encapsulated EOs against food infesting bacteria, fungi, and associated toxins. The improved antimicrobial potential of nanoencapsulated EOs has been ascribed to controlled released of bioactives and easy access to food regions supporting the microbial proliferation rendered by the subcellular size of the emulsionic particles. The antimicrobial mechanism of action of encapsulated EO was attributed to the inhibitory action on ergosterol biosynthesis, release of biologically important ions including calcium, potassium, and magnesium, 260 and 280 nm absorbing materials, loss in ATP pool caused by disturbances in proton motive force, and oxidation of biomolecules caused by ROS. The antimicrobial activity of nanoencapsulated EOs has been reported to be influenced by fabrication process parameters including pH, temperature, concentration, and chemical composition of polymer matrix and tripolyphosphate content, homogenization speed, time of sonication, surfactant, and most notably the chemical characteristics of natural products employed.

Although, the EOs and non-encapsulated formulation thereof has achieved great success in preventing the growth of food spoilage bacteria, fungi, and microbial toxins, the commercialized formulations for application in food industries are still lacking. Most importantly, a future potential model with emphasis on the release and delivery of bioactive components/constituents from nanoemulsion needs to be evaluated before practical application in food and agricultural industries. The time taken to release the volatile component greatly depends on the concentration of wall material. In the case of lipid carriers, low-fat product displayed burst release of the volatile component, while, high fat gave sustained-release (Dasgupta and Ranjan, 2018). Release of the non-volatile component occurs due to simple dilution. After nanoemulsion dilution, some of the bioactive components could move from oil droplets to the aqueous phase which may be considered as an efficient release mechanism of non-volatiles (Maher et al., 2015). A number of intrinsic and extrinsic factors *viz*. pH, dilution, ion strength, enzyme activity, and temperature determine the release rate of bioactives in the nanoemulsion system. Interestingly, a decrease in particle size in the nanoemulsion system regulates the oil-water partition coefficient which further affects the bioavailability and release profile. Walia et al. (2017) demonstrated encapsulation efficiency, loading capacity, and viscosity of vitamin-D is a prime factor for stable and efficient release from nanoemulsion. Additionally, droplets encapsulated into hydrogel particles increased the path length for diffusion and minimized the release rate (Komaiko and McClements, 2014). Some of the important future research opportunities linked with the application of EO based nanoformulation:

(a) Fabrication of nanoemulsions with enhanced stability under fluctuating conditions during food processing.
(b) Development of nanoemulsions with improved nanoencapsulation efficiency and loading capacity.
(c) The fate and transport of nanoemulsions in the natural environment needs to be extensively studied in order to safeguard environmental homeostasis.
(d) The search for newer wall materials as well as the design of composite materials having suitability for food applications to encapsulate the EOs is of immense importance in food industries.
(e) Appraisal of the cost-benefit ratio for the fabrication of nanoemulsion could be helpful in the development of cost-effective formulations.
(f) The effect on gut microflora is an important aspect while thinking about the supplementation of EO-based nanoemulsion to prevent the spoilage of food and toxin secretion by bacterial and fungal pathogens.
(g) As extremely small-sized nanoemulsions may get rapid access to human cellular systems and may exert undesirable effects after consumption of treated food, extensive *in vivo* and *in vitro* investigations should be conducted to avoid undesirable effects on human health.

Current challenges and research gaps of nanoencapsulated bioactive components for practical applications are:

(a) Minimal use of chemical substances during encapsulation.
(b) Long-lasting controlled release of encapsulated bioactive compounds.
(c) Use of chief plant-based polymeric matrix for encapsulation, and
(d) Maximization of loading of bioactive compounds in encapsulating matrix.

## Author Contributions

ND and AD conceptualized the idea of the manuscript and supervised the writing of the manuscript. AM wrote the manuscript, designed the figures and table along with contributions from VS, JP, and AK. VS, SD, and NU did the manuscript editing, formatting and gave the final approval for its submission in its present form. All the authors contributed to the article and approved the submitted version.

## Conflict of Interest

The authors declare that the research was conducted in the absence of any commercial or financial relationships that could be construed as a potential conflict of interest.

## Publisher’s Note

All claims expressed in this article are solely those of the authors and do not necessarily represent those of their affiliated organizations, or those of the publisher, the editors and the reviewers. Any product that may be evaluated in this article, or claim that may be made by its manufacturer, is not guaranteed or endorsed by the publisher.
