# Supplementary material for: Essential Oil Nanoemulsion as Eco-Friendly and Safe Preservative: Bioefficacy Against Microbial Food Deterioration and Toxin Secretion, Mode of Action, and Future Opportunities
Source: Front Microbiol. 2021 Nov 29;12:751062. doi: 10.3389/fmicb.2021.751062 (PMC8667777; doi:10.3389/fmicb.2021.751062)
Supplement: Supplementary file 1 [file Table_1.pdf]

Table 1 Antibacterial efficacy of EOs nanoemulsion in foods

S. No.	EO/Component nanoemulsion	Effective constitutes	Biopolymer	Fabrication Method	Effective over/ Test organism	Key findings	References
**Gram (+)ve**							
**1**	*Piper nigrum*	β -caryophyllene, limonene, δ-3-carene and pinene	Hydroxypropyl β-cyclodextrin	Inclusion complex	*Staphylococcus aureus*	• Minimum Inhibitory Concentration (MIC): MIC value of Nanoemulsion of, black pepper was improved 4 times against the *S. aureus* (from 1000 to 250 µg/mL).<br>• Effective mechanism: Encapsulated oil primarily acted on cell membrane and inside the cytoplasm.	Rakmai et al. (2017)

**2**	*Eugenia caryophyllata*	Eugenol and β - caryophyllene	Chitosan	Ionic gelation	*S. aureus and L. monocytogenes*	• Minimum inhibitory volume (MIV) of oil loaded nanoparticle is 2 µL against *S. aureus* and *L. monocytogenes*<br>• Effective mechanism: Nano-sized Clove EO can penetrate bacterial cell wall and destroyed cell membrane.	Hadidi et al. (2020)
**3**	*Origanum vulgare*<br><br>*Thymus capitatus*	Thymol, γ - terpinene, and Cymene<br>Carvacrol, *p*-Cymene, β – caryophyllene, and γ - terpinene	Polymeric poly (ε-caprolactone)	Nanoprecipitation		• MIC of *Thymus* nanoemulsion against *S. aureus* and *L. monocytogenes* was 0.5 and 0.25 mg/mL, respectively.<br>• In case of *O. vulgare* nanoemulsion MIC was 0.5 and 1 mg/mL against *S. aureus* and *L. monocytogenes*, respectively.	Granata et al. (2018)

						• Effective mechanism: Effective constituents of EO encapsulated in polycaprolactone able to interact with outer cell membrane, increasing permeability and fluidity, which causes structural and functional damage to cell membrane.	
**4**	*Moringa oleifera*	Palmitic acid and phytol	Chitosan	Ionic gelation		• *M. oleifera* nanoemulsion acted as promising antibacterial agent against *S. aureus* and *L. monocytogenes* at 4 °C and 25 °C during 10 days storage of cheese without affecting	Lin et al. (2019)

						organoleptic properties.<br>• Effective mechanism: After treatment bacteria with encapsulated oil disturbed the cell membrane resulting the leakage of cell contents.	
**5**	d-Limonene	-	ε -polylysine (ε-PL)	High pressure Homogenization	*S. aureus Bacillus subtilis* and *Saccharomyces cerevisiae*	• d-limonene nanoemulsion encapsulated into ε-PL resulted in synergistic antimicrobial activity against the wide range Gram +ve and Gram –ve bacteria.	Zahi et al. (2017)
**Gram (-)ve**							
**6**	*Piper nigrum*	**-**	Hydroxypropyl β-cyclodextrin	Inclusion complex		• Minimum Inhibitory Concentration (MIC): Improvement in MIC	Rakmai et al. (2017)

					Escherichia coli	value from against E. coli from 2000 to 500 µg/mL.<br>• Effective mechanism- Encapsulated oil primarily acted on cell membrane and cytoplasm	
**7**	*Thymus daenensis*	-	Tween 80 (2% w/w), and lecithin (0.001% w/w)	Sonication		• Minimum Inhibitory Concentration (MIC): The nanoemulsion showed effective inhibitory action against *E. coli* with MIC 0.4 mg/mL.<br>• Effective mechanism:The formulation inhibited the *E. coli* population by enhancing mechanism of action, bringing the EOs into	Moghimi et al. (2016b)

						close proximity with the plasma membrane.	
**8**	*Thymus vulgaris*	Thymol, para-cymene, linalool, terpinene,α-pinene, and carvacrol	Casein and maltodextrin	Spray drying	.	• Minimum Inhibitory Concentration (MIC): Encapsulated EO showed 0.1 mg/mL MIC value against *S. typhimurium*, *L. monocytogens*, *S. aureus* and *E. coli*.<br>• Effective mechanism: Encapsulated EO showed higher antimicrobial activity in comparison to thyme EO through slow release of volatile components that protected by wall materials during *in situ* storage of hamburger like meat product.	Radünz et al. (2020)

**9**	*Utrica dioica*	Carvacrol, carvone, naphthalene, and anethol	Chitosan	Ionic gelation		• The nanoencapsulated nettle EO boosted the antibacterial activity against the foodborne bacteria *viz. S. aureus*, *L. monocytogenes*, *S. typhi*, *E. coli*, and *B, cereus*	Bagheri et al. (2021)
**10**	*Melaleuca alternifolia*	Terpinene-4-ol, γ-terpinene, and terpinene	β-cyclodextrin	Inclusion complex		• TTO/ β-CD inclusion complex enhanced the shelf life of beef against *E. coli* and inhibited 99.99% food spoilage bacteria at 4 °C over 7 days storage.	Cui et al. (2018)
**11**	*Cinnamomum zeylanicum*	Cinnamaldehyde	Medium-chain triglyceride	Emulsification		• MIC value of cinnamaldehyde nanoemulsion was 98 µg/mL against *E. coli*.<br>• Effective mechanism: Encapsulation of cinnamaldehyde	Tian et al. (2016)

						showed strong inhibitory effect against *E. coli* within 9 h of treatment.	
**12**	Cinnamon oil	Eugenol and cinnamaldehyde	Coconut oil, and Tween 80	Microfluidization and Ultrasonication		• Nanoemulsion showed promising application in food sector against the *E.coli*.	Yildirim et al. (2017)
**13**	Cinnamaldehyde	-	Chitosan	Ionic gelation	*Pseudomonas aeruginosa*	• MIC of cinnamaldehyde nanoemulsion against	Subhaswaraj et al. (2018)

						P. aeruginosa was found to be 1000 µg/mL.<br>• Cinnamaldehyde nanoemulsion inhibited quorum sensing activity and prevented food spoilage with controlled as well as slow release at specific sites.	
**14**	Cinnamon oil	Cinnamaldehy deand α-pinene	Chitosan and whey protein	Nanoprecipitati on	*E. coli*,<br>*P. fragi*, and *Shewanella putrefaciens*	• Antibacterial activity of cinnamon oil emulsion against *S. aureus* was observed at 0.041%.<br>• Cinnamon oil emulsion acted as strong antimicrobial and long-lasting biological molecule	Yang et al. (2021)

						against various food contaminating Gram +ve and Gram –ve bacteria indicating utilization as potential preservative in food industry.	
**15**	*Rosmarinus officinalis*	-	Chitosan (CS) and benzoic acid (BA)	Sonication	*Salmonella typhimurium*	• Nanogel of *R. officinalis* showed the MIC value 5 µg/mL against the *S. typhimurium*.<br>• Nanogel of *R. officinalis* applied on beef cutlet at the rate of 0.5 mg/g had positive effects on the color and shelf-life.	Hadian et al. (2017)
**16**	*Cinnamodendron dinisii*	1,8-cineole	Chitosan and Zein	Nanoprecipitation	*S. typhimurium* and *Shigella flexneri*	• Nanoemulsion of *C. dinisii* film showed greater antimicrobial activity against Gram	Xavier et al. (2021)

						–ve bacteria and stabilized the deterioration reaction in ground beef leading to prolong shelf-life under refrigerated storage.	
**17**	Lime oil	-	Chitosan	Nanoprecipitation	*Shigella dysenteriae*	• Chitosan nanoparticles incorporating lime EO performed highest antibacterial activity against *S. dysenteriae* and inhibition halo (HI) value was 3.5 cm at 40 µL volume.	Sotelo-Boyás et al. (2017)

Table 2. Antifungal efficacy of EOs nanoemulsion in food

S. No.	Effective over/ Test organism	EO/Component (nanoemulsion)	Effective constituents	Biopolymer	Method	Key findings	References
**1**	*A. flavus*	*Origanum majorana*	Terpinen-4-ol, α-terpineol, linalool, and caryophyllene	Chitosan	Ionic gelation	• MIC of *O. majorana* EO nanoemulsion was observed to be 1µL/mL against *A. flavus*.<br>• Effective mechanism: *O. majorana* EO nanoemulsion showed remarkable inhibitory efficacy against the *A. flavus* growth and AFB_1 production as compared to free *O. majorana* EO by enhancing reduction of ergosterol concentration.	Chaudhari et al. (2020)
**2**		*Eugenia caryophyllata*	Eugenol and caryophyllene	Chitosan	Sonication	• *E. caryophyllata* EO nanoemulsion showed remarkable antifungal (0.25 µL/mL) and aflatoxin B_1	Kujur et al. (2021)

						inhibitory (0.15 μL/mL) activity.<br>• *E. caryophyllata* EO nanoemulsion exhibited promising fungitoxic, aflatoxin B_1 inhibitory activity and preserved the sensory properties of six month stored maize seeds.	
**3**		*Cuminum cyminum*	-	Chitosan and Caffeic acid	Sonication	• Encapsulated *C. cyminum* EO showed promising MIC (350 ppm) against *A. flavus* under sealed condition as compared to free oil (650 ppm).	Zhaveh et al. (2015)
**4**		Orange oil	-	β-cyclodextrin	Inclusion complex	• Orange EO nanoemulsion showed complete inhibition at 900 mg/mL.<br>• Nanoencapsulated EO improved the antimicrobial activity in contaminated cakes	Kringel et al. (2021)

**5**		*Cananga odorata*	Linalool and benzyl acetate	Chitosan	Ionic gelation	• *C. odorata* EO nanoemulsion reduced the *A. flavus* growth and aflatoxin B_1 secretion at 1.0 and 0.75 µL/mL, respectively.	Upadhyay et al. (2021)
**6**		Eugenol	-	Chitosan	Ionic gelation	• Eugenol nanoemulsion showed antifungal and aflatoxin B_1 inhibitory activity at 0.07 and 0.06 µL/mL concentration, respectively.<br>• Eugenol-loaded chitosan nanoemulsion significantly protected the rice seeds from fungal, AFB_1 contamination, lipid peroxidation and maintained their mineral contents and macronutrients	Das et al. (2021a)
**7**	*A. niger*	*Eugenia caryophyllata*	Eugenol	Chitosan	Ionic gelation	• Inhibition of *A. niger* mycelial growth was 1.5 mg/mL.	Hasheminejad et al. (2019)

**8**		Cinnamon leaf oil	Eugenol, benzyl benzoate, caryophyllene, and aceteeugenol	Whey protein	High pressure homogenization	• 0.25 µg/mL concentration of CEO inhibited 75% fungal population, whereas, the same concentration in nanoemulsion form was noted to completely inhibit *A. niger* population.	Ribes et al. (2017)
**9**	*A. parasiticus*	*Schinus molle*	D-limonene, o-cimene, β-phellandrene, δ-cadinene and caryophillene	Chitosan	Ionotropic gelation	• Nanoencapsulated *S. molle* EO at 500 µg/mL concentration showed strong antifungal and antiaflatoxigenic activity.	López-Meneses et al. (2018)
**10**		*Schinus molle*	D-limonene, β-phellandrene,and o-cimene	Chitosan	Nanoprecipitation	• *S. molle* EO nanoemulsion effectively reduced (40-50%) viable spores of *A. parasiticus.* • Antifungal effect of emulsion due to cytoplasmic coagulation, protoplast leakage and loss of conidiation.	Luque-Alcaraz et al. (2016)

**11**	*Alternaria alternata*	*Origanum vulgare*	α-pinene, β-myrcene, carvacrol, borneol, caryophyllene, γ-terpinene, ρ-cymene and terpinolene	Chitosan	Electrospraying deposition	• Oil loaded chitosan emulsion showed antifungal activity associated with destruction of cell membrane permeability as well as remarkable depolarization of membrane.<br>• Oregano EO loaded nanoemulsion can be used in agriculture and food industry as a promising fungicidal agent against *A. alternata.*	Yilmaz et al. (2019)
**12**		α-pinene	-	Chitosan	Nanoprecipitation	• Nanoemulsion inhibited *A. alternata* growth during the cold storage of bell peppers and maintained the physiochemical property	Hernández-López et al. (2020)
**13**	*Penicillium italicum P. digitatum and*	*Litsea cubeba*	Neral and limonene	β-cyclodextrin	Inclusion complex	• MIC values of *L. cubeba* EO nanoemulsion was found to be 3.13, 1.56, and 0.78 mg/mL against *P. italicum*, *P.*	Wang et al. (2020)

	Geotrichum citri-aurantii					*digitatum* and *G. candidum*, respectively.<br>• *L. cubeba* EO nanoemulsion exhibited antifungal activity and preserved citrus fruits from postharvest decay and disease.	
**14**	*P. chrysogenum and*<br>*A. flavus*	*Ocimum basilicum*	Methyl chavicol and linalool	Saponin	Ultrasonication	• The nanoemulsion of basil oil had higher antifungal activity against food spoilage fungi. Nanoemulsion at 1000 ppm concentration displayed 64-67% inhibitory activity over *P. chrysogenum and A. flavus*.	Gundewadi et al. (2018)
**15**	*Fusarium graminearum*	*Cymbopogon martini*	Geraniol, geranial, geranyl propionate and geranyl acetone	Chitosan	Ionic gelation	• *C. martini* EO nanoemulsion completely inhibited the growth of *F. graminearum* and mycotoxins at 700 and 900 ppm.<br>• *C. martini* EO nanoemulsion showed remarkable	Kalagatur et al. (2018)

						antifungal and antimycotoxigenic activity against *F. graminearum* during 28 days storage of maize grain.	

Table 3. Application of nanoencapsulated EO in active packaging of food products

S. No.	Essential oil or their active ingredients	Packaging types/ Emulsifier/Carrier	Food products	Type of microorganism	Biological activity	Reference
1	Citral	Tween 80/Sodium alginate	Pineapple fruit	*Salmonella enterica S. typhimurium* and *L. monocytogenes*	• Sodium alginate based edible coating of different concentration of citral nanoemulsion i.e. 0.1% (NE1), 0.5% (NE2), and 1% (NE3) significantly reduced the microbial load of fresh cut pineapples during storage for 12 days.<br>• High concentration of citral in NE3 led to improvement in the sensory quality and texture of pineapple, thus the NE2 could be explored as for commercial application.	Prakash et al. (2020)
2	Carvacrol	Tween 80/ Starch Polymer/Montmorillonite	Vegetables and fruits	*E. coli*	• Carvacrol nanoemulsion showed strong antimicrobial efficacy in food packaging	de Souza et al. (2020)

					against *E. coli* due to destabilization and partial destruction of bacterial cell membrane, electron transport, respiration and permeability.	
3	Linalool	Tween 80	Pineapple fruit	*S. typhimurium*	• Nanoemulsion biofilm on the surface of cut pineapple effectively reduced the *S. typhimurium* and increased the shelf life of cut pineapple.<br>• Nanoemulsion enhanced twofold antibacterial activity against *S. typhimurium* by increasing ability to cell membrane integrity.	Prakash et al. (2019)
4	*Origanum virens*	Whey protien	Sausages	*L. monocytogenes*	• Coating of Portuguese sausage delayed the lipid peroxidation and inhibited the growth of *L. monocytogenes* during storage condition.<br>• EO coating enhanced the antibacterial activity of oil via	Catarino et al. (2017)

					sustained release as well as increased the solubility of oil.	
5	*Rosmarinus officinalis*	Chitosan/ ε-Poly-L-lycine	Carbonado chicken	All types of microorganism viz. Coliform, molds and Yeast	• Total bacterial count reduced effectively by nanoemulsion coating and enhances the shelf life of carbonado chicken meat during 6 days storage.<br>• Similar results found in case of Yeast and molds.	Huang et al. (2020)
6	*Zataria multiflora* and *Bunium persucum*	Tween 80/ Chitosan	Turkey fillet	*S. enteritidis* and *L. monocytogens*	• Shelf life of turkey fillet could be extended to 9 days by using nanoemulsion containing oil during 20 days storage at refrigeration condition.<br>• Turkey meat coated with nanoemulsion of *Zataria multiflora* EO (1%) showed best antibacterial activity.	Keykhosravy et al. (2020)
7	*Thymus vulgaris*	Tween 80/ Calcium alginates	Ground beef	*Staphylococcus aureus*, *E. coli*, Yeast and Molds	• Active biopolymer films revealed high antimicrobial activity in agar test agar	Almasi et al. (2021)

					diffusion tests, when apply to package ground beef.	
8	Lemmon grass oil	Tween 80/ Chitosan	Grape berries	*S. typhimurium*	• The nanoemulsion coating showed greater efficiency in microbial safety against *Salmonella* and preserving ready-to-eat grape berries.	Oh et al. (2017)
9	*Ferulago angulate*	Gelatin-Chitosan	Turkey Meat	*S. aureus* and Coliform	• Turkey meat packed with gelatin-chitosan film containing 0.5% *Ferulago angulate* EO reduced the microbial growth and enhances the shelf life of preserved the turkey meat.	Naseri et al. (2020)
10	*Cuminum cyminum* and *Piper nigrum*	Tween 80	Fruits and vegetables	*E. coli*, and *S. enterica*	• Cumin and pepper oil nanoemulsion inhibited the bacterial population on fruits and vegetable by forming biofilm.<br>• MIC of Cumin oil nanoemulsion against *E. coli*,	Amrutha et al. (2017)

					and *S. enterica* was 100 and 80 µL/mL receptively.<br>• MIC of Pepper oil against *E. coli*, and *S. enterica* was 120 and 140 µL/mL, receptively.	
11	*Eryngium campestre*	Tween 80/Chitosan	Cherry fruit	Mesophilic aerobic bacteria	• Coating on cherries decreased the microbial population and weight loss compared to control during storage.<br>• Coating enhanced the firmness, pH, antioxidant activity and total phenolic content.	Arabpoor et al. (2021)
12	Thymol	Poly-(lactic) acid/poly (butylene-succinateco-adipate) (PLA/PBSA) blend film	Bread	*Aspergillus* spp. and *Penicillium* spp.	• The antifungal activity of thymol coated film was very efficacious against the *Aspergillus* and *Penicillium* spp. in both packed bread and *in vitro* condition.	Suwanamornlert et al. (2020)
13	Cinnamon Oil	Tween 80	Bread	*P. digitatum*	• The film having 1.5 and 3% Cinnamon oil showed no	Fasihi et al. (2019)

		/Carboxymethyl cellulose/polyvinyl alcohol			mycelial growth of *P. digitatum* over 60 days incubation of bread.<br>• Active coating at 0.5, 1.5 and 3% exhibited complete fungal growth inhibition on bread slices.<br>• Thus, coating showed the higher effectivity in comparison to films during inhibition of fungal growth in bread slices.	
14	*Origanum vulgare*	Tween 20/ Basil Seed Gum (BSG)	Apricot	Yeast and Molds	• Coating significantly improved the EO dispersibility.<br>• Coatings of BSG + 6% *Origanum* oil was showed the most effective in reducing yeast and molds population, extending the shelf life of apricot cuts.	Hashemi et al. (2017)

15	*Mentha piperata*	Chitosan	Grape fruits	*A. niger*, *Botrytis cinerea*, *P. expansum* and *R. stolonifer*	• The combined effect of chitosan and *Mentha* oil effectively delayed the rate of grape decay during storage at room and low temperature.<br>• Coatings of grape barriers strongly reduced the mold infections by increasing antibacterial activity of coatings.	Guerra et al. (2016)
16	Cinnamon Oil	Chitosan	Cherry Fruits	*P. citrinum* and *A. flavus*	• Cinnamon oil improved the growth inhibition of *P. citrinum* from 33.17 mm to 36.23 mm with increasing concentration from 1% to 4% respectively.<br>• Similar result obtained in case of *A. flavus*. Growth inhibition from 31.12 mm to 35.28 mm with increasing concentration from 1% to 4% respectively.	Xing et al. (2016)

17	Lemmon grass oil	Flaxseed Gum (FSG)	Pomegranate Arils	Yeast and Molds	• The coating enhanced the potential of oil to enter the cell and improves the interaction between cell and oil.<br>• The coatings significantly reduce the number of microbes and maintain the intrinsic colour of pomegranate arils.	Yousuf, and Srivastava (2017)

Supplementary references:

Almasi, L., Radi, M., Amiri, S., and McClements, D. J. (2021). Fabrication and characterization of antimicrobial biopolymer films containing essential oil-loaded microemulsions or nanoemulsions. Food Hydrocoll., 117, 106733. https://doi.org/10.1016/j.foodhyd.2021.106733

Amrutha, B., Sundar, K., and Shetty, P. H. (2017). Spice oil nanoemulsions: Potential natural inhibitors against pathogenic E. coli and *Salmonella* spp. from fresh fruits and vegetables. LWT., 79, 152-159. https://doi.org/10.1016/j.lwt.2017.01.031

Arabpoor, B., Yousefi, S., Weisany, W., and Ghasemlou, M. (2021). Multifunctional coating composed of *Eryngium campestre* L. essential oil encapsulated in nano-chitosan to prolong the shelf-life of fresh cherry fruits. Food Hydrocoll., 111, 106394. https://doi.org/10.1016/j.foodhyd.2020.106394

Bagheri, R., Ariaii, P., and Motamedzadegan, A. (2021). Characterization, antioxidant and antibacterial activities of chitosan nanoparticles loaded with nettle essential oil. J. Food Meas. Charact. 15(2), 1395-1402. https://doi.org/10.1007/s11694-020-00738-0

Catarino, M. D., Alves-Silva, J. M., Fernandes, R. P., Gonçalves, M. J., Salgueiro, L. R., Henriques, M. F., et al., (2017). Development and performance of whey protein active coatings with *Origanum virens* essential oils in the quality and shelf life improvement of processed meat products. Food Cont., 80, 273-280. https://doi.org/10.1016/j.foodcont.2017.03.054

Chaudhari, A. K., Singh, V. K., Das, S., Prasad, J., Dwivedy, A. K., and Dubey, N. K. (2020). Improvement of in vitro and in situ antifungal, AFB_1 inhibitory and antioxidant activity of *Origanum majorana* L. essential oil through nanoemulsion and recommending as novel food preservative. Food Chem. Toxicol. 143, 111536. https://doi.org/10.1016/j.fct.2020.111536

Cui, H., Bai, M., and Lin, L. (2018). Plasma-treated poly (ethylene oxide) nanofibers containing tea tree oil/beta-cyclodextrin inclusion complex for antibacterial packaging. Carbohydr. Polym. 179, 360-369. https://doi.org/10.1016/j.carbpol.2017.10.011

Das, S., Singh, V. K., Dwivedy, A. K., Chaudhari, A. K., and Dubey, N. K. (2021a). Eugenol loaded chitosan nanoemulsion for food protection and inhibition of Aflatoxin B1 synthesizing genes based on molecular docking. Carbohydr. Polym. 255, 117-339. https://doi.org/10.1016/j.carbpol.2020.117339

de Souza, A. G., Dos Santos, N. M. A., da Silva Torin, R. F., and dos Santos Rosa, D. (2020). Synergic antimicrobial properties of Carvacrol essential oil and montmorillonite in biodegradable starch films. Int. J. Biol. Macromol., 164, 1737-1747. https://doi.org/10.1016/j.ijbiomac.2020.07.226

Fasihi, H., Noshirvani, N., Hashemi, M., Fazilati, M., Salavati, H., and Coma, V. (2019). Antioxidant and antimicrobial properties of carbohydrate-based films enriched with cinnamon essential oil by Pickering emulsion method. Food Packag. Shelf Life., 19, 147-154. https://doi.org/10.1016/j.fpsl.2018.12.007

Granata, G., Stracquadanio, S., Leonardi, M., Napoli, E., Consoli, G. M. L., Cafiso, V., et al. (2018). Essential oils encapsulated in polymer-based nanocapsules as potential candidates for application in food preservation. Food Chem. 269, 286-292. https://doi.org/10.1016/j.foodchem.2018.06.140

Guerra, I. C. D., de Oliveira, P. D. L., Santos, M. M. F., Lúcio, A. S. S. C., Tavares, J. F., Barbosa-Filho, J. M., et al., (2016). The effects of composite coatings containing chitosan and *Mentha* (piperita L. or x villosa Huds) essential oil on postharvest mold occurrence and quality of table grape cv. Isabella. Innov Food Sci Emerg Technol., 34, 112-121. https://doi.org/10.1016/j.ifset.2016.01.008

Gundewadi, G., Sarkar, D. J., Rudra, S. G., and Singh, D. (2018). Preparation of basil oil nanoemulsion using *Sapindus mukorossi* pericarp extract: Physico-chemical properties and antifungal activity against food spoilage pathogens. Ind. Crops Prod. 125, 95-104. https://doi.org/10.1016/j.indcrop.2018.08.076

Hadian, M., Rajaei, A., Mohsenifar, A., and Tabatabaei, M. (2017). Encapsulation of *Rosmarinus officinalis* essential oils in chitosan-benzoic acid nanogel with enhanced antibacterial activity in beef cutlet against *Salmonella typhimurium* during refrigerated storage. LWT, 84, 394-401. https://doi.org/10.1016/j.lwt.2017.05.075

Hadidi, M., Pouramin, S., Adinepour, F., Haghani, S., and Jafari, S. M. (2020). Chitosan nanoparticles loaded with clove essential oil: Characterization, antioxidant and antibacterial activities. Carbohydr. Polym. 236, 116-075. https://doi.org/10.1016/j.carbpol.2020.116075

Hashemi, S. M. B., Khaneghah, A. M., Ghahfarrokhi, M. G., and Eş, I. (2017). Basil-seed gum containing *Origanum vulgare* subsp. viride essential oil as edible coating for fresh cut apricots. Postharvest Biol. Technol., 125, 26-34. https://doi.org/10.1016/j.postharvbio.2016.11.003

Hasheminejad, N., Khodaiyan, F., and& Safari, M. (2019). Improving the antifungal activity of clove essential oil encapsulated by chitosan nanoparticles. Food Chem. 275, 113-122. https://doi.org/10.1016/j.foodchem.2018.09.085

Hernández-López, G., Ventura-Aguilar, R. I., Correa-Pacheco, Z. N., Bautista-Baños, S., and Barrera-Necha, L. L. (2020). Nanostructured chitosan edible coating loaded with α-pinene for the preservation of the postharvest quality of *Capsicum annuum* L. and *Alternaria alternata* control. Int. J. Biol. Macromol., 165, 1881-1888. https://doi.org/10.1016/j.ijbiomac.2020.10.094

Huang, M., Wang, H., Xu, X., Lu, X., Song, X., and Zhou, G. (2020). Effects of nanoemulsion-based edible coatings with composite mixture of rosemary extract and ε-poly-L-lysine on the shelf life of ready-to-eat carbonado chicken. Food Hydrocoll., 102, 105576. https://doi.org/10.1016/j.foodhyd.2019.105576

Kalagatur, N. K., Nirmal Ghosh, O. S., Sundararaj, N., and Mudili, V. (2018). Antifungal activity of chitosan nanoparticles encapsulated with *Cymbopogon martinii* essential oil on plant pathogenic fungi *Fusarium graminearum*. Front. pharmacol. 9, 610. https://doi.org/10.3389/fphar.2018.00610

Keykhosravy, K., Khanzadi, S., Hashemi, M., and Azizzadeh, M. (2020). Chitosan-loaded nanoemulsion containing *Zataria multiflora* Boiss and *Bunium persicum* Boiss essential oils as edible coatings: Its impact on microbial quality of turkey meat and fate of inoculated pathogens. Int. J. Biol. Macromol., 150, 904-913. https://doi.org/10.1016/j.ijbiomac.2020.02.092

Kringel, D. H., Lang, G. H., Dias, Á. R. G., Gandra, E. A., Valente Gandra, T. K., and da Rosa Zavareze, E. (2021). Impact of encapsulated orange essential oil with β-cyclodextrin on technological, digestibility, sensory properties of wheat cakes as well as *Aspergillus flavus* spoilage. J. Sci. Food Agric. https://doi.org/10.1002/jsfa.11211

Kujur, A., Kumar, A., and Prakash, B. (2021). Elucidation of antifungal and aflatoxin B_1 inhibitory mode of action of *Eugenia caryophyllata* L. essential oil loaded chitosan nanomatrix against *Aspergillus flavus*. Pestic. Biochem. Physiol. 172, 104755. https://doi.org/10.1016/j.pestbp.2020.104755

Lin, L., Gu, Y., and Cui, H. (2019). Moringa oil/chitosan nanoparticles embedded gelatin nanofibers for food packaging against *Listeria monocytogenes* and *Staphylococcus aureus* on cheese. Food Packag. Shelf Life. 19, 86-93. https://doi.org/10.1016/j.fpsl.2018.12.005

López-Meneses, A. K., Plascencia-Jatomea, M., Lizardi-Mendoza, J., Fernández-Quiroz, D., Rodríguez-Félix, F., Mouriño-Pérez, R. R., et al., (2018). *Schinus molle* L. essential oil-loaded chitosan nanoparticles: Preparation, characterization, antifungal and anti-aflatoxigenic properties. LWT, 96, 597-603. https://doi.org/10.1016/j.lwt.2018.06.013

Luque-Alcaraz, A. G., Cortez-Rocha, M. O., Velázquez-Contreras, C. A., Acosta-Silva, A. L., Santacruz-Ortega, H. D. C., Burgos-Hernández, A., et al. (2016). Enhanced antifungal effect of chitosan/pepper tree (*Schinus molle*) essential oil bionanocomposites on the viability of *Aspergillus parasiticus* spores. J. Nanomater. 2016. https://doi.org/10.1155/2016/6060137

Moghimi, R., Ghaderi, L., Rafati, H., Aliahmadi, A., and McClements, D. J. (2016b). Superior antibacterial activity of nanoemulsion of *Thymus daenensis* essential oil against E. coli. Food chem. 194, 410-415. https://doi.org/10.1016/j.foodchem.2015.07.13

Naseri, H. R., Beigmohammadi, F., Mohammadi, R., and Sadeghi, E. (2020). Production and characterization of edible film based on gelatin–chitosan containing *Ferulago angulate* essential oil and its application in the prolongation of the shelf life of turkey meat. J. Food Process. Preserv., 44(8), e14558. https://doi.org/10.1111/jfpp.14558

Oh, Y. A., Oh, Y. J., Song, A. Y., Won, J. S., Song, K. B., and Min, S. C. (2017). Comparison of effectiveness of edible coatings using emulsions containing lemongrass oil of different size droplets on grape berry safety and preservation. LWT, 75, 742-750. https://doi.org/10.1016/j.lwt.2016.10.033

Prakash, A., Baskaran, R., and Vadivel, V. (2020). Citral nanoemulsion incorporated edible coating to extend the shelf life of fresh cut pineapples. LWT, 118, 108851. https://doi.org/10.1016/j.lwt.2019.108851

Prakash, A., Vadivel, V., Rubini, D., and Nithyanand, P. (2019). Antibacterial and antibiofilm activities of linalool nanoemulsions against *Salmonella typhimurium*. Food Biosci., 28, 57-65. https://doi.org/10.1016/j.fbio.2019.01.018

Radünz, M., dos Santos Hackbart, H. C., Camargo, T. M., Nunes, C. F. P., de Barros, F. A. P., Dal Magro, J., et al., (2020). Antimicrobial potential of spray drying encapsulated thyme (*Thymus vulgaris*) essential oil on the conservation of hamburger-like meat products. Int. J. Food Microbiol. 330, 108-696. https://doi.org/10.1016/j.ijfoodmicro.2020.108696

Rakmai, J., Cheirsilp, B., Mejuto, J. C., Torrado-Agrasar, A., and Simal-Gándara, J. (2017). Physico-chemical characterization and evaluation of bio-efficacies of black pepper essential oil encapsulated in hydroxypropyl-beta-cyclodextrin. Food Hydrocoll. Food hydrocolloids, 65, 157-164. https://doi.org/10.1016/j.foodhyd.2016.11.014

Ribes, S., Fuentes, A., Talens, P., Barat, J. M., Ferrari, G., and Donsì, F. (2017). Influence of emulsifier type on the antifungal activity of cinnamon leaf, lemon and bergamot oil nanoemulsions against *Aspergillus niger*. Food Control. 73, 784-795. https://doi.org/10.1016/j.foodcont.2016.09.044

Sotelo-Boyás, M. E., Correa-Pacheco, Z. N., Bautista-Baños, S., and Corona-Rangel, M. L. (2017). Physicochemical characterization of chitosan nanoparticles and nanocapsules incorporated with lime essential oil and their antibacterial activity against food-borne pathogens. LWT, 77, 15-20. https://doi.org/10.1016/j.lwt.2016.11.022

Subhaswaraj, P., Barik, S., Macha, C., Chiranjeevi, P. V., and Siddhardha, B. (2018). Anti quorum sensing and anti biofilm efficacy of cinnamaldehyde encapsulated chitosan nanoparticles

against *Pseudomonas aeruginosa* PAO1. LWT. 97, 752-759. https://doi.org/10.1016/j.lwt.2018.08.011

Suwanamornlert, P., Kerddonfag, N., Sane, A., Chinsirikul, W., Zhou, W., and Chonhenchob, V. (2020). Poly (lactic acid)/poly (butylene-succinate-co-adipate) (PLA/PBSA) blend films containing thymol as alternative to synthetic preservatives for active packaging of bread. Food Packag. Shelf Life., 25, 100515. https://doi.org/10.1016/j.fpsl.2020.100515

Tian, W. L., Lei, L. L., Zhang, Q., and Li, Y. (2016). Physical stability and antimicrobial activity of encapsulated cinnamaldehyde by self-emulsifying nanoemulsion. J. Food Process Eng., 39(5), 462-471. https://doi.org/10.1111/jfpe.12237

Upadhyay, N., Singh, V. K., Dwivedy, A. K., Chaudhari, A. K., and Dubey, N. K. (2021). Assessment of nanoencapsulated *Cananga odorata* essential oil in chitosan nanopolymer as a green approach to boost the antifungal, antioxidant and in situ efficacy. Int. J. Biol. Macromol. 171, 480-490. https://doi.org/10.1016/j.ijbiomac.2021.01.024

Wang, Y., Yin, C., Cheng, X., Li, G., Yang, S., and Zhu, X. (2020). β-Cyclodextrin inclusion complex containing *Litsea cubeba* essential oil: Preparation, optimization, physicochemical, and antifungal characterization. Coatings, 10(9), 850. https://doi.org/10.3390/coatings10090850

Xavier, L.O., Sganzerla, W.G., Rosa, G.B., da Rosa, C.G., Agostinetto, L., de Lima Veeck, A.P., et al., 2021. Chitosan packaging functionalized with *Cinnamodendron dinisii* essential oil loaded zein: A proposal for meat conservation. Int. J. Biol. Macromol. 169, pp.183-193. https://doi.org/10.1016/j.ijbiomac.2020.12.093

Xing, Y., Xu, Q., Yang, S. X., Chen, C., Tang, Y., Sun, S., et al., (2016). Preservation mechanism of chitosan-based coating with cinnamon oil for fruits storage based on sensor data. Sensors., 16(7), 1111. https://doi.org/10.3390/s16071111

Yang, K., Liu, A., Hu, A., Li, J., Zen, Z., Liu, Y., et al., 2021. Preparation and characterization of cinnamon essential oil nanocapsules and comparison of volatile components and antibacterial ability of cinnamon essential oil before and after encapsulation. Food Control. 123, p.107783. https://doi.org/10.1016/j.foodcont.2020.107783

Yildirim, S. T., Oztop, M. H., and Soyer, Y. (2017). Cinnamon oil nanoemulsions by spontaneous emulsification: Formulation, characterization and antimicrobial activity. LWT. 84, 122-128. https://doi.org/10.1016/j.lwt.2017.05.041

Yilmaz, M. T., Yilmaz, A., Akman, P. K., Bozkurt, F., Dertli, E., Basahel, A., et al., (2019). Electrospraying method for fabrication of essential oil loaded-chitosan nanoparticle delivery systems characterized by molecular, thermal, morphological and antifungal properties. Innov Food Sci Emerg Technol . 52, 166-178. https://doi.org/10.1016/j.ifset.2018.12.005

Yousuf, B., and Srivastava, A. K. (2017). Flaxseed gum in combination with lemongrass essential oil as an effective edible coating for ready-to-eat pomegranate arils. Int. J. Biol. Macromol., 104, 1030-1038. https://doi.org/10.1016/j.ijbiomac.2017.07.025

Zahi, M. R., El Hattab, M., Liang, H., and Yuan, Q. (2017). Enhancing the antimicrobial activity of d-limonene nanoemulsion with the inclusion of ε-polylysine. Food chem. 221, 18-23. https://doi.org/10.1016/j.foodchem.2016.10.037

Zhaveh, S., Mohsenifar, A., Beiki, M., Khalili, S. T., Abdollahi, A., Rahmani-Cherati, T., et al., (2015). Encapsulation of *Cuminum cyminum* essential oils in chitosan-caffeic acid nanogel with enhanced antimicrobial activity against Aspergillus flavus. Ind. Crops Prod. 69, 251-256. https://doi.org/10.1016/j.indcrop.2015.02.028
